# Healthcare Service Access and Utilization among Syrian Refugees in Turkey

**DOI:** 10.5334/aogh.2353

**Published:** 2019-03-20

**Authors:** Ismail Tayfur, Mücahit Günaydin, Selim Suner

**Affiliations:** 1 University of Health Sciences, Faculty of Medicine, TR; 2 Giresun University, Faculty of Medicine, TR; 3 Brown University, The Warren Alpert Medical School, US

## Abstract

**Background Aim:**

Many Syrian civil war refugees are housed in carefully designed camps, however 60% of all refugees in host countries reside outside of specifically designated camps. Turkey hosts the largest number of refugees displaced in the civil war of Syria. In the present study, we aimed to illustrate healthcare services provided to Syrian refugees in Turkey.

**Method:**

Data presented in this retrospective observational registry study was obtained from multiple sources including official websites and written communications contributed by Turkish Disaster and Emergency Management Agency(AFAD), Turkish Ministry of Health and Turkish Red Crescent as well as the United Nations High Commissioner for Refugees(UNHCR). The number of refugee camps, total number of refugees housed in these camps, the demographic characteristics of the refugees and a breakdown of social and healthcare services provided in the camps including the number of healthcare professionals serving in the refugee camps were analyzed.

**Results:**

According to data from UNHCR as of January 12, 2017, there are 4,904,021 registered refugees from Syria of which 2,854,968 (58.2%) are residing in Turkey. Organized health care services specifically for Syrian refugees in Turkey was first established in April 29, 2011 in Hatay. Preventative health services are also provided for Syrian refugees. Among refugees living in the camps 25% and 33% were not vaccinated for polio and measles respectively. The percentage of unvaccinated refugees living outside the camps were even higher for these viruses (45% for polio and 41% for measles). This poses a public health threat for the population where these refugees reside.

**Conclusion:**

One of the major concerns for countries hosting refugees from Syria is the introduction of infectious diseases. Of the major deficiencies in refugee health care has been preventative services. Within refugee camps, preventative services for pregnant woman, newborns and young children as well as family planning services are not at optimal levels. These services are even more restricted for refugees living outside of established camps. There have also been shortcomings in the care of the elderly and those with chronic health problems.

## Introduction

The Arab Spring, an uprising of the populous in multiple Middle Eastern countries, started with the self-immolation of a young street merchant on December 17, 2010 in Tunisia. Months of demonstrations, riots and regime change ensued in multiple nations [1]. In Syria, the government forces resisted change, leading to a civil war, which continues and has resulted in the largest refugee crisis of this century. On April 29, 2011, 252 Syrian refugees fled over barbed wire fences to seek asylum in the Hatay province of Turkey. The Turkish government granted their asylum request and announced that they supported the people of Syria. This action led to a large flow of refugees from Syria along the 911 km border with Turkey. When the civil war started in Syria, the population was 22.5 million. The latest reports show that nearly 50% of the population is now internally or externally displaced [2]. Lebanon, Jordan and Iraq, which also border Syria, started accepting refugees in 2011 and 2012. Refugees were initially housed in camps, but many have spilled out and 60% of refugees are currently residing in host countries outside of specifically designated camps. In this study we aim to illustrate the healthcare services provided to Syrian refugees in Turkey, which is the country hosting the largest number of people displaced in the wake of the civil war in Syria.

## Methods

In this retrospective study data presented was obtained from multiple sources including documents and statistics found on official websites and written communications from AFAD (Turkish Disaster and Emergency Management Agency), Turkish Ministry of Health, Turkish Red Crescent, as well as the UNHCR(United Nations High Commissioner for Refugees). A written inquiry was also made to AFAD for information not contained in these sources, such as the number of refugee camps, total number of refugees housed in these camps, the demographics of the refugees and a breakdown of social and healthcare services provided in the camps including the number of healthcare professionals serving in the refugee camps, commonly encountered medical conditions, number of cases in emergency clinics, their provisional diagnoses and the number of patients transferred to tertiary care hospitals. Data was analysed with Statistical Package for Social Sciences (SPSS) ver. 17,0. Values are given as mean and percentage.

## Results

**Figure 1:**
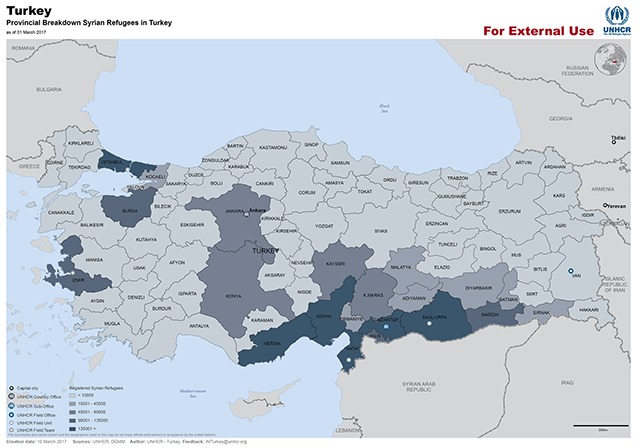
The distrubution of refugees within Turkey as of March 10, 2017.

**Figure 2:**
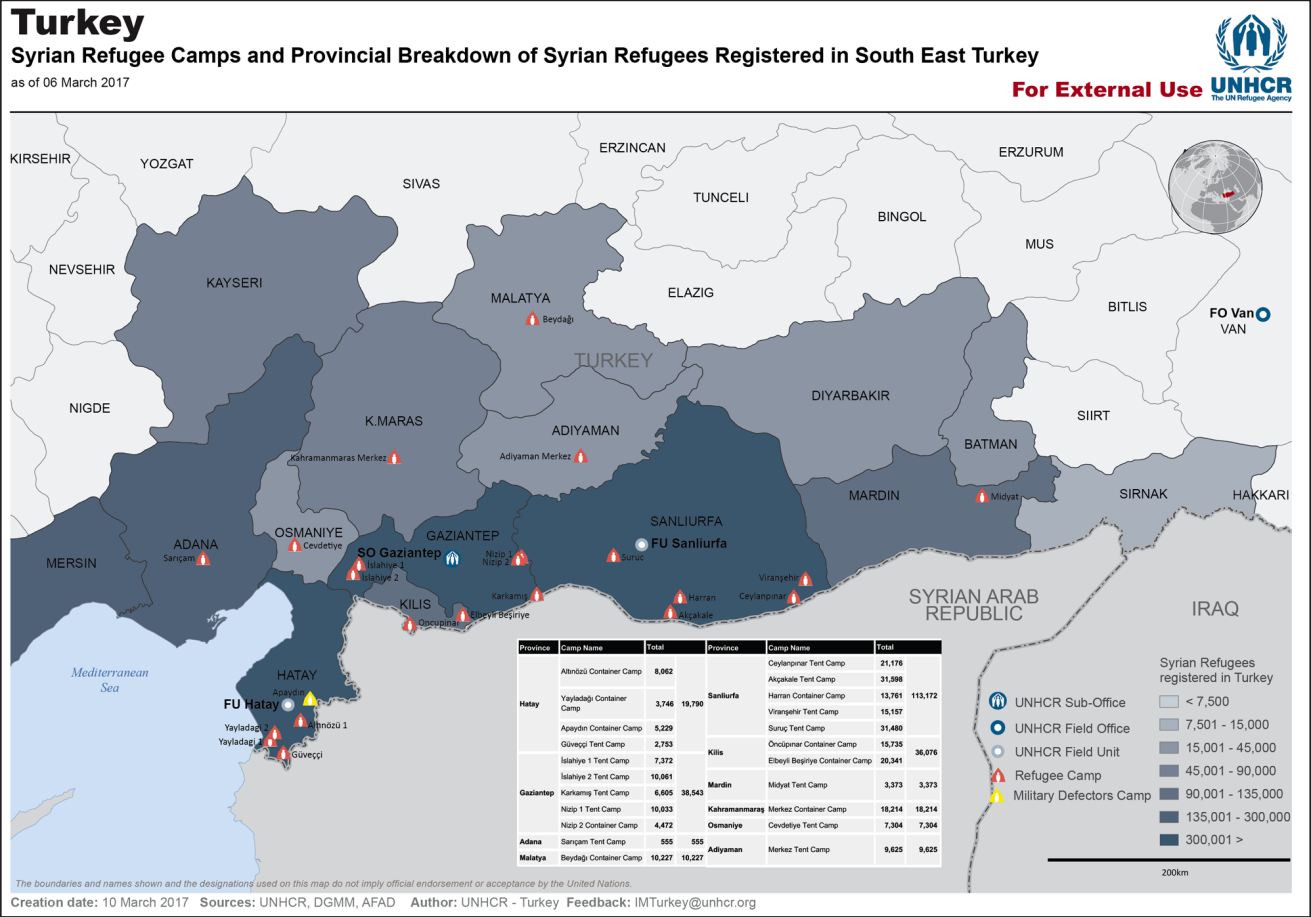
The location of refugee camps along the Syrian-Turkey border.

According to data from UNHCR as of January 12, 2017, there are 4,904,021 registered refugees from Syria of which 2,854,968 (58.2%) are residing in Turkey. There are also 1,017,433 (20.7%) refugees in Lebanon, 655,496 (13.4%) in Jordan, 230,836 (4.7%) in Iraq and 116,013 (2.4%) in Egypt. The remaining 0.6% are distributed among other countries including western developed nations such as Germany, USA and Canada. Of the refugees in Turkey 53.2% are male, 44% under the age of 18 (13.7% under the age of five years) [3]. The distribution of refugees within Turkey as of March 10, 2017 is shown in Figure 1. While there is a concentration of refugees in rural areas near the Syrian border, there are also a considerable number of refugees in large cities such as Istanbul, remote from the border [4]. The location of refugee camps along the Syrian-Turkey border is shown in Figure 2. The distribution of refugees is summarized in Table 1 [4].

**Table 1:** The distribution by country of the number of registered refugees.

Country	Number of Refugees

TURKEY	2,739,326
LEBANON	1,048,275
JORDAN	655,217
IRAQ	247,339
EGYPT	117,702
NORTH AFRICA	29,275
**TOTAL**	**4,837,134**

**Figure 3:**
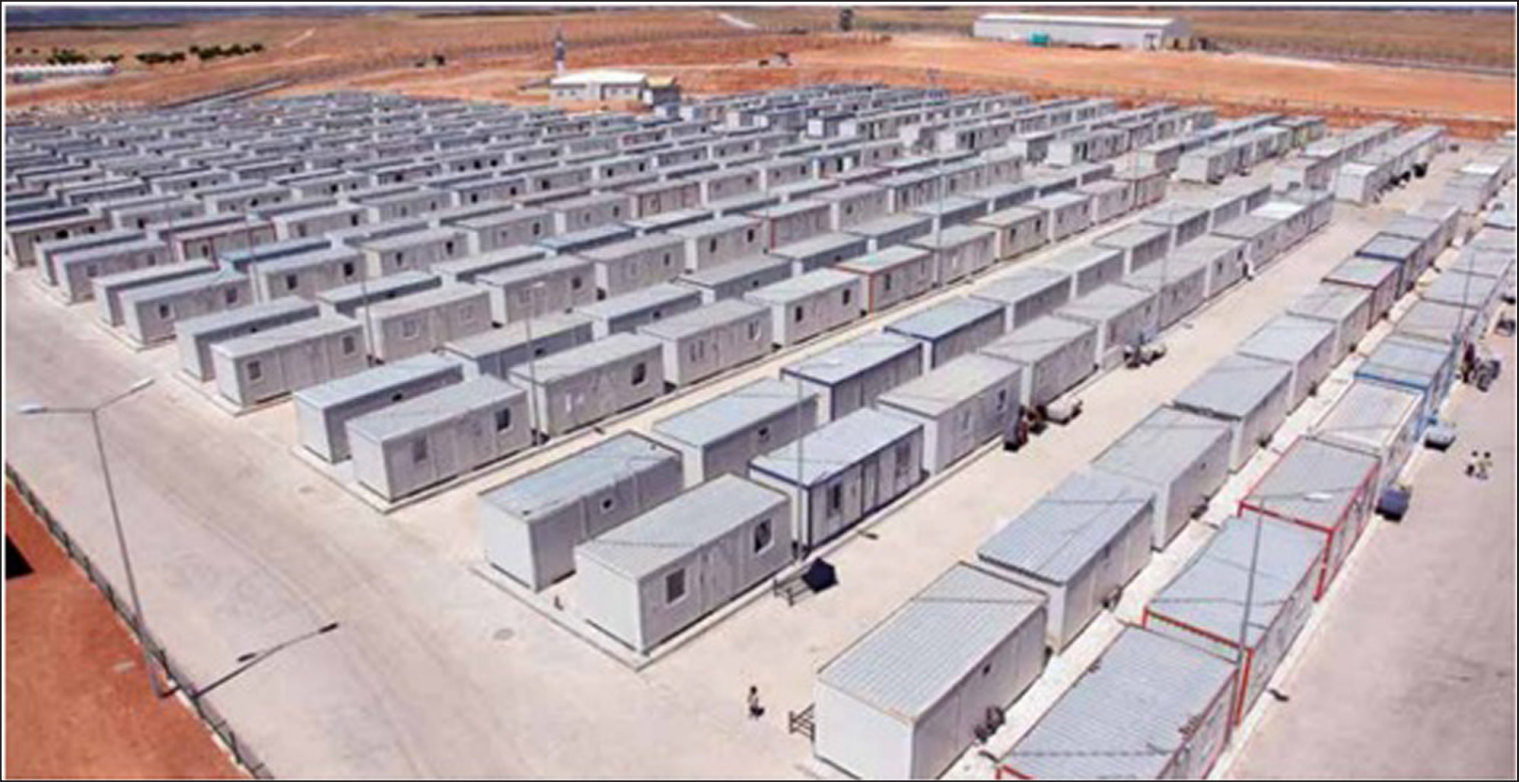
A typical refugee camp erected for Syrian refugees.

**Figure 4:**
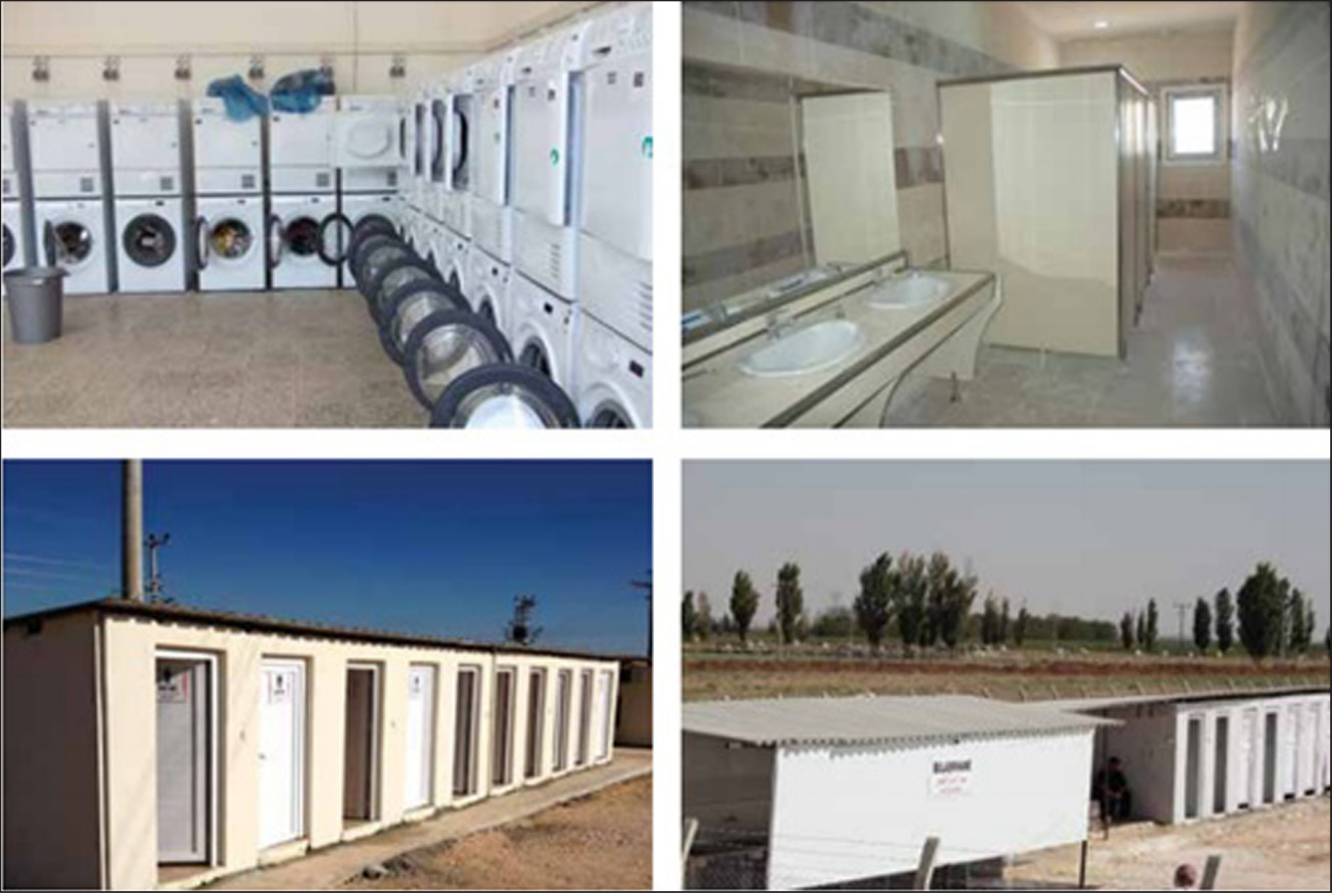
Refugee camps in Turkey, also include separate bathing and laundry facilities for men and women.

Healthcare for Syrian refugees in Turkey was first established in April 29, 2011 in Hatay. Refugee camps were strategically located in safe locales and include amenities for healthy living. The camps were designed to conform to or exceed the Sphere guidelines [https://www.sphereproject.org/]. Figure 3 is a photograph depicting a typical refugee camp erected for Syrian refugees [5]. Each inhabitant of the camp was provided with 3 liters of drinking water and 50 liters of potable water for personal hygiene, cleaning and cooking per day using a water distribution system easily accessible by all inhabitants. Camps also include separate bathing and laundry facilities for men and women (1 shower stall per 25 inhabitants, 1 washing machine per 20 inhabitants) as depicted in Figure 4. Camp management, security, logistics and distribution centers and mobile hospitals were located centrally within the camps. Firefighting units were placed in close proximity to the camps and the healthcare facility was designed as a separate 27,000 ft^2^ facility within each camp.

**Figure 5:**
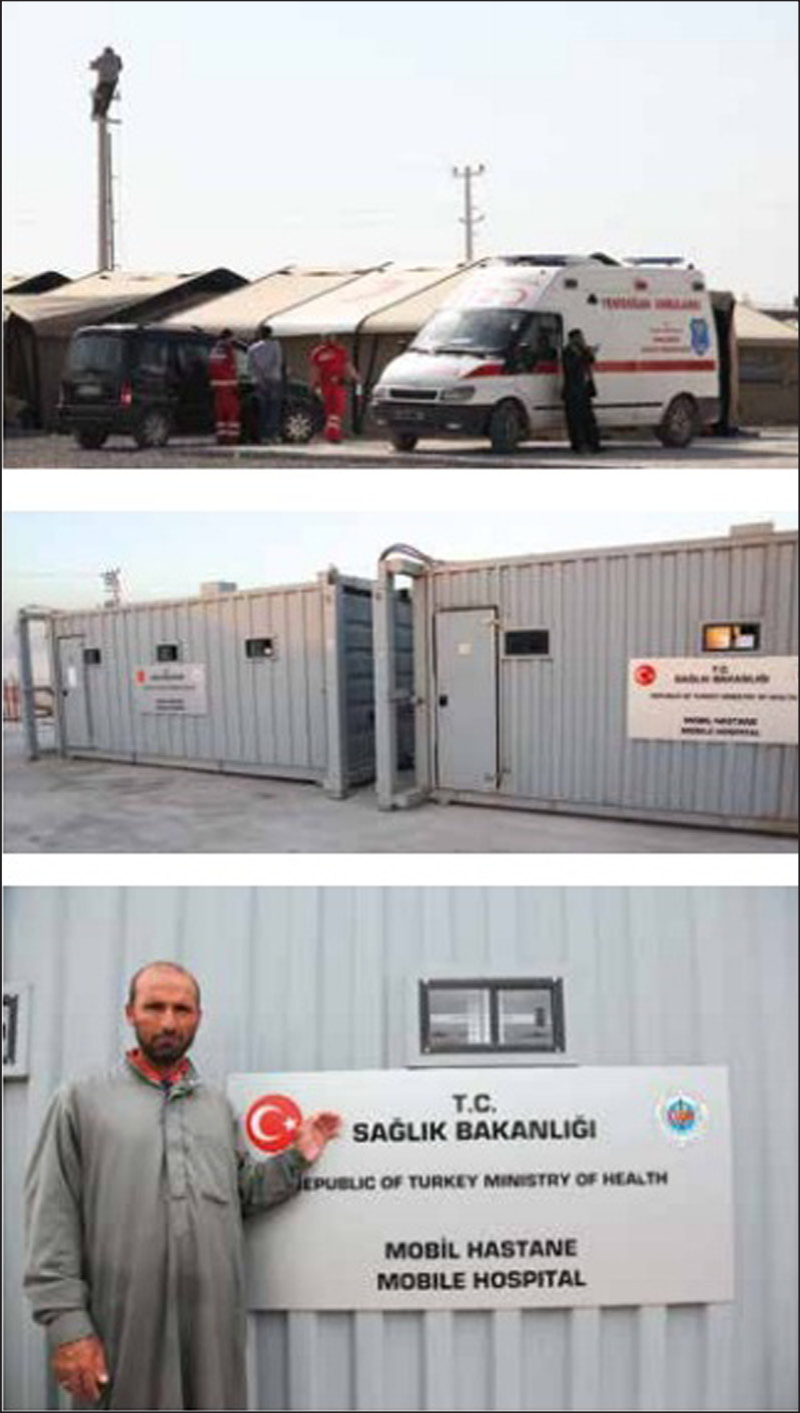
Camp management, security, logistics and distribution centers and mobile hospitals were located centrally within the camps.

As of November 7, 2016 there were 21 health care facilities with 71 specialist and 110 general practitioner physicians, 30 dentists, 102 health aids in 26 refugee camps distributed among 10 cities (Figure 5) [6]. Between 2011 and 2016 there were 784,570 visits to secondary hospitals and 30,860 patients transferred to tertiary centers for treatment. In the same time period, there were 666,418 surgical procedures conducted at secondary receiving hospitals and 20,443 operative cases in tertiary centers. There were also 140,968 regular and 3647 high-risk deliveries performed. Among this population, there were 4882 deaths recorded in secondary hospitals and 1396 in tertiary centers.

Between 2011 and April 2016, 31,590 injured refugees were transported to area hospitals from the Syria-Turkey border utilizing 75 ambulances. In addition, there were nine teams equipped to respond to CBRNE (chemical, biological, radiological, nuclear and explosives) incidents stationed at three locations along the border. These teams were equipped with all necessary equipment and received extensive training to perform decontamination and medical treatment of casualties from a CBRNE event.

In addition, preventative health services were also provided for Syrian refugees. Between 2011 and 2016, 1,804,574 refugees were vaccinated. Health maintenance was provided for 284,395 infants, 242,822 children and 91,921 pregnant women. During the same period, surveillance efforts revealed 112,576 cases of watery diarrhea, 54 cases of bloody diarrhea, 1313 cases of Hepatitis A and 1,033,226 cases of respiratory infections in all of the refugee camps. Among refugees living in the camps 25% and 33% were not vaccinated for polio and measles respectively. The percentage of unvaccinated refugees living outside the camps was even higher for these viruses (45% for polio and 41% for measles). This poses a public health threat for the population where these refugees reside [7].

## Conclusions

During the current humanitarian crisis arising from the civil conflict in Syria, Turkey is harboring 56% of the externally displaced population. The government of Turkey has undertaken extraordinary measures to ensure the humanitarian rights of this population and has provided shelter, food, education and healthcare to this vulnerable population.

One of the major concerns for countries hosting refugees from Syria is the introduction of infectious diseases. Polio, cutaneous Leishmaniasis, Typhoid, and Hepatitis A are endemic in Syria [8]. With the exception of polio, the other diseases are also seen in Turkey. In fact, living in large camps places the refugees at risk for contracting infectious disease, particularly multi-drug resistant tuberculosis, meningitis, rabies, pneumonia and bronchitis [8].

One of the major deficiencies in refugee health care has been preventative services. Within the camps preventative services for pregnant woman, newborns and young children, as well as family planning services, are not at optimal levels. These services are even more restricted for refugees living outside of established camps [9]. There have also been shortcomings in the care of the elderly and those with chronic health problems. There have been delays in care for refugees requiring dialysis, cancer treatment and physical therapy.

Those refugees living in established camps are able to seek healthcare in camp clinics. Those requiring advanced services are transferred to outside hospitals and in some cases to regional tertiary care centers. Registered refugees living outside of camps are able to seek healthcare services at family practice primary care services, Syrian Refugee Health Clinics and any Ministry of Health Hospitals. Patients are then transferred to private hospitals or University Health Centers for tertiary care if required. Refugees have a 20% co-pay on cost of medications. All other health expenses, including hospitalization and surgical care are covered [10].

In a 2014 retrospective study evaluating the health records of 251 ambulatory Syrian refugee patients, the most frequent healthcare visit was through the emergency department and the most frequent encounter was for respiratory illness. Cost of care in this population was $48 per patient and 12,031 dollars and 93 cents total [11]. According to data from the AFAD, the expenditure for Syrian refugees in Turkey has exceeded $12 billion, whereas international aid was $512 million [12].

The difficulty in providing healthcare to refugees from the Syrian crisis is not unique to Turkey. In March of 2011, the number of refugees from Syria in Lebanon was roughly 30% of the Lebanese population. The healthcare system in Lebanon, which was already in crisis mode, has been decimated by the arrival of refugees despite significant international assistance [13]. In a study conducted on 1376 Syrian refugees in Lebanon, the baseline rates of non-infectious chronic illness (diabetes, hypertension, vascular heart disease, arthritis and respiratory illness) was evaluated and compared to the Lebanese population. This study showed that while only chronic respiratory illness was more prevalent among the refugee population, nearly half of the study population suffered from one of the chronic diseases causing a significant burden on the healthcare system [14].

Doocy et al. evaluated 1550 Syrian refugees living outside established camps in Jordan. They showed that hypertension was the most prevalent non-infectious chronic disease among refugees, followed by arthritis, diabetes, respiratory illness and cardiac disease. They also noted that nearly 50% of this population had a chronic health condition. The investigators showed that most of this population was not getting care for their chronic illness. They specified that the major reason for this was cost for healthcare services [15].

In Europe, the number of Syrian refugees is very low compared to those in Turkey, Lebanon and Jordan. Between April 2011 and September 2016, the total number of refugee applications in Europe was 884,461. Of these applications 64% was for Germany and Sweden; 22% for Hungary, Austria, Holland, Denmark and Bulgaria; and 14% for other European Union nations [16].

A study evaluating the health records of 3907 Syrian refugees in Belgium between September and November 2015 showed traumatic injury in 11%. Injury surpassed respiratory illness, dental, skin and gastrointestinal related complaints. This was thought to be secondary to a long and arduous journey to reach Belgium [17].

The Greek islands in the Mediterranean are the gateway for many Syrian refugees into Europe. A study on the refugee population in the Greek islands showed that healthcare was expensive, sporadic and mostly provided by non-governmental organizations. The most common health issues encountered in this study were infectious diseases, traumatic injuries and mental health issues [18].

While there are many shortcomings in the provision of healthcare for Syrian refugees in Turkey, which is hosting the largest number, studies have shown that problems in the delivery of care are encountered in all countries harboring refugees. The causes for this include lack of preparedness among the healthcare and insurance systems to respond to the surge of patients and the added cost of care. Despite all hurdles, the government in Turkey has stepped up and provided a system to deal with the enormous surge in healthcare needs as a result of the unprecedented number of refugees from the Syrian crisis.

## Abbreviations

**UNHCR:** United Nations High Commissioner for Refugees.

**AFAD:** Turkish Disaster and Emergency Management Agency.

**CBRNE:** Chemical-Biological-Radiological-Nuclear-Explosives.
